# Antibody-dependent enhancement representing in vitro infective progeny virus titer correlates with the viremia level in dengue patients

**DOI:** 10.1038/s41598-021-91793-0

**Published:** 2021-06-11

**Authors:** Atsushi Yamanaka, Hisham Ahmed Imad, Weerapong Phumratanaprapin, Juthamas Phadungsombat, Eiji Konishi, Tatsuo Shioda

**Affiliations:** 1 Mahidol-Osaka Center for Infectious Diseases, Faculty of Tropical Medicine, Mahidol University, 420/6 Ratchawithi Road, Ratchathewi, Bangkok, 10400 Thailand; 2 Mahidol-Osaka Center for Infectious Diseases, Research Institute for Microbial Diseases, Osaka University, 3-1 Yamada-oka, Suita, Osaka 565-0871 Japan; 3 Department of Clinical Tropical Medicine, Faculty of Tropical Medicine, Mahidol University, 420/6 Ratchawithi Road, Ratchathewi, Bangkok, 10400 Thailand; 4 BIKEN Endowed Department of Dengue Vaccine Development, Faculty of Tropical Medicine, Mahidol University, 420/6 Ratchawithi Road, Ratchathewi, Bangkok, 10400 Thailand; 5 Department of Viral Infections, Research Institute for Microbial Diseases, Osaka University, 3-1 Yamada-oka, Suita, Osaka 565-0871 Japan

**Keywords:** Dengue virus, Viral host response

## Abstract

Dengue virus (DENV) causes dengue fever (DF) and dengue hemorrhagic fever in humans. Some DF patients suddenly develop severe symptoms around the defervescent period. Although the pathogenic mechanism of the severe symptoms has not been fully elucidated, the viremia level in the early phase has been shown to correlate with the disease severity. One of the hypotheses is that a phenomenon called antibody-dependent enhancement (ADE) of infection leads to high level of viremia. To examine the plausibility of this hypothesis, we examined the relationship between in vitro ADE activity and in vivo viral load quantity in six patients with dengue diseases. Blood samples were collected at multiple time points between the acute and defervescent phases, and the balance between neutralizing and enhancing activities against the autologous and prototype viruses was examined. As the antibody levels against DENV were rapidly increased, ADE activity was decreased over time or partially maintained against some viruses at low serum dilution. In addition, positive correlations were observed between ADE activity representing in vitro progeny virus production and viremia levels in patient plasma samples. The measurement of ADE activity in dengue-seropositive samples may help to predict the level of viral load in the subsequent DENV infection.

## Introduction

Dengue virus (DENV), belonging to the family *Flaviviridae*, genus *Flavivirus*, is distributed throughout tropical and subtropical areas of the world^1^. DENV is transmitted by *Aedes* mosquito species and causes dengue fever (DF) and severe dengue in humans. Approximately 3.9 billion people are under the risk of infection^2^. An estimated 390 million people are infected with DENV annually, and 100 million of these individuals show clinical symptoms^3^. Therefore, dengue is one of the most important mosquito-borne viral diseases worldwide, and it should be controlled to the greatest extent possible.

The four serotypes of DENV (DENV-1, DENV-2, DENV-3 and DENV-4) are genetically distinct, and there is complicated immunological cross-reactivity among them^4^. Secondary heterotypic infection has been epidemiologically demonstrated to increase the risk of severe forms—namely, dengue hemorrhagic fever (DHF) and dengue shock syndrome (DSS)^5^. Patients showing dengue with warning signs have a risk of developing disease severity, with the emergence of severity usually occurring around the defervescence phase, beginning at days 3–7 of illness^6,7^. The mortality rate of cases with DSS is much higher than that of cases without DSS^8^. Although a mechanism associated with the severity and a surrogate marker predicting the deterioration have not been fully identified yet, high levels of viremia have been shown to be related to disease severity^6,9–13^. Moreover, a recently published meta-analysis revealed that there was an association between disease severity and viremia duration^14^. On the other hand, some studies have reported finding no association between disease severity and high viremia levels^15,16^. Antibody-dependent enhancement of infection (ADE) that increases the viremia level has been proposed as one of the pathogenic mechanisms in DHF/DSS^17^; in the case of ADE the increase occurs by viral internalization via Fc gamma receptors^18^. Recently, a potential relationship between ADE and human disease severity in DENV infection has been reported^19^. However, it is still unclear whether in vitro ADE can be used for the prediction in subsequent clinical outcomes.

Enhancing antibodies (EAbs), which exclusively play a role of the ADE phenomenon^20^, may be associated with an increase in the viremia level in DENV infection^21,22^. In contrast, neutralizing antibodies (NAbs) have a biological function to decrease the viremia level to protect the host from DENV infection^23^, while most NAbs show ADE activity at subneutralizing doses^24^. These functional antibodies are supposed to be introduced by one of three routes: (i) DENV infection, (ii) maternal antibody from a DENV-seropositive mother and (iii) other flavivirus infection. We have previously demonstrated that a DENV-immune serum (polyclonal form) could be represented with a cocktail of functional monoclonal EAbs and NAbs^25^. Therefore, the balance activity between EAbs and NAbs might be critical to control the outcome (protection or pathogenesis). We previously developed a simple method to detect the balance between the enhancing and neutralizing activities^26^, and demonstrated that mouse monoclonal EAbs and NAbs competed over the neutralizing activities in vitro^25,27^. Specifically, the neutralizing activity of an NAb was reduced in the presence of a sufficient level of an EAb, suggesting that the relative capacity for neutralization might be easily affected by the balance between NAbs and EAbs.

In the present study, we evaluated the balance between neutralizing and enhancing activities in sera collected from dengue patients at multiple time points between the acute and defervescent phases. The six autologous viruses isolated from the respective patients were used as assay antigens, allowing us to examine the balance antibody assay with autologous combinations between patient sera and virus antigens. We also measured the number of viral RNA copies in plasma samples collected at multiple time points, and revealed a correlation between the in vitro ADE activity and in vivo viral load quantity.

## Results

### Balance between the neutralizing and enhancing activities against autologous viruses

**Figure 1:**
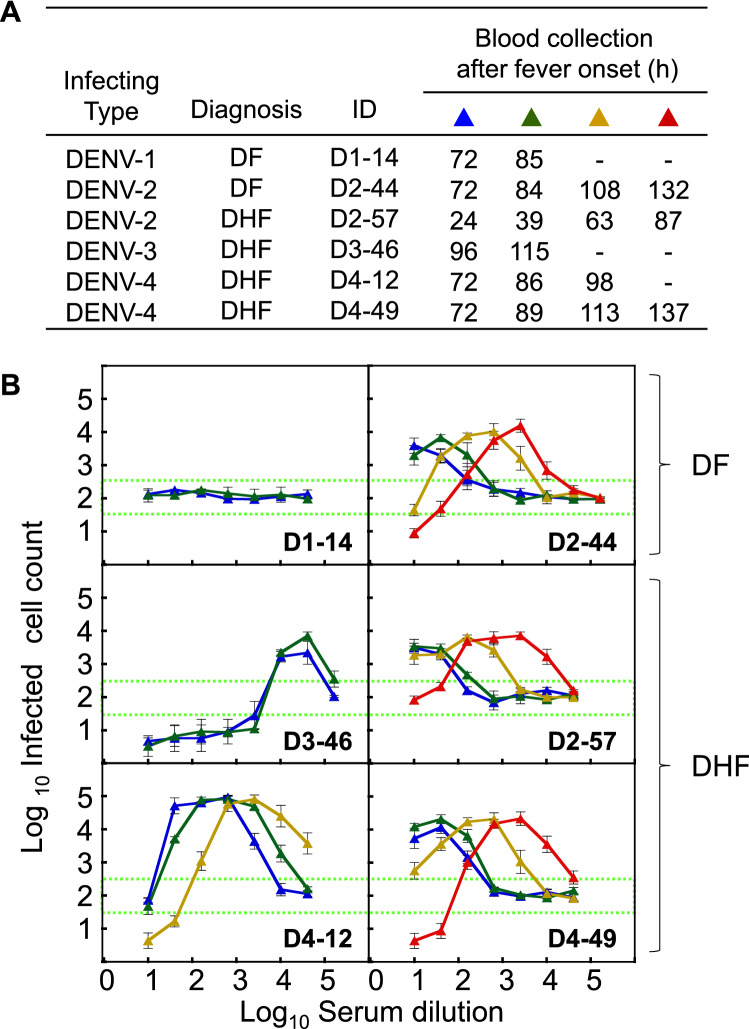
Balance between neutralizing and enhancing activities against autologous virus. (**A**) Demographic data for the six dengue patients enrolled in this study. Blood samples were collected on the day of hospitalization and the following time points at 12–24 h intervals until a maximum 137 h after fever onset. (**B**) NAb/EAb balance activity against the autologous virus. The NAb/EAb-balance assay was performed with a combination of autologous virus and serum. The *blue*, *green*, *yellow* and *red* triangles, corresponding to (**A**), indicate the hours after fever onset. The abscissa indicates the serum dilutions and the ordinate shows the numbers of infected cells (both expressed as log10). Each data point represents the average of two separate assays; error bars indicate the SDs. Dotted lines indicate the mean numbers of infected cells plus or minus three times the SD (mean ± 3SD), to define neutralizing or enhancing activity, respectively.

Six hospitalized adult patients, who had been enrolled in the previous study^13^, were recruited into the present study, and an autologous DENV clinical strain was successfully isolated from each of them. The demographic information (infecting serotype, diagnosis [DF or DHF], ID number and sample collection period [h] after the fever onset) is shown in Fig. 1A. Serum samples, which were collected at several time points between the acute and defervescent phases, were subjected to an antibody assay to determine the balance between neutralizing and enhancing activities (NAb/EAb-balance assay) using each autologous virus. As shown in Fig. 1B, five patients—i.e., all of the enrolled patients except for patient D1-14—showed dose (dilution)-dependent antibody activity patterns displaying neutralizing and enhancing activities. Since the abscissa indicates serum dilutions, increases in antibody levels over time would cause the dose-dependent antibody activity curve to shift from the left to the right. Therefore, the comparison of the antibody activity curves indicated an increase in antibody levels. Antibody titers determined by a conventional neutralization test support an evidence of the shifts with time gaining (Table 1), indicating that their neutralizing antibody titers rapidly increased by 6 ~ 78-fold during the early disease stage. The increase of their antibody levels (up to 64-fold) was confirmed by the enzyme-linked immunosorbent assay (ELISA), too (Supplementary Table S1). Similar dose-dependent patterns were observed in patients D2-44, D2-57 and D4-49, even though these patients had different infecting serotypes and diagnoses. On the other hand, patients D1-14 and D3-46 did not show a remarkable shift during the observation period, as neutralizing antibody titers showed < twofold increase (Table 1). As a patient D1-14 was classified as primary infection by IgG/IgM immunochromatography tests in the previous study^13^, no functional antibody activity was observed in 72 and 85 h samples. Although patient D3-46 showed strong neutralization with a wide range of low-middle serum dilutions (1:10 ~ 1:2560) (Fig. 1B), this patient was diagnosed with DHF with severe clinical symptoms.

**Table 1:** Neutralizing antibody titers against the autologous virus.

	Hours	Patient ID					
		D1-14	D2-44	D2-57	D3-46	D4-12	D4-49
Period after fever onset	24	–	–	1:73	–	–	–
	39	–	–	1:150	–	–	–
	63	–	–	1:331	–	–	–
	72	1:70^a^	1:234	–	–	1:188	1:38
	84	–	1:1912	–	–	–	–
	85	1:62	–	–	–	–	–
	86	–	–	–	–	1:260	–
	87	–	–	1:2867	–	–	–
	89	–	–	–	–	–	1:104
	96	–	–	–	1:25,553	–	–
	98	–	–	–	–	1:1173	–
	108	–	1:1213	–	–	–	–
	113	–	–	–	–	–	1:417
	115	–	–	–	1:46,414	–	–
	132	–	1:3500	–	–	–	–
	137	–	–	–	–	–	1:2952
Infection history		Primary	Secondary	Secondary	Secondary	Secondary	Secondary

^a^Neutralizing antibody titer was expressed as the maximum serum dilution showing > 75% focus reduction (FRNT75).

### Balance between neutralizing and enhancing activities against four prototype viruses

**Figure 2:**
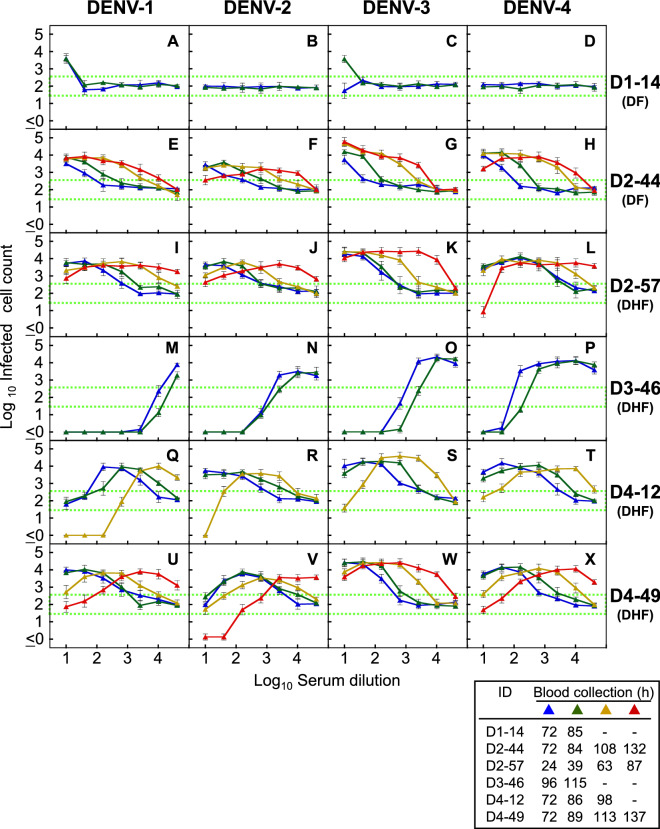
Balance between neutralizing and enhancing activities against four prototype viruses. The NAb/EAb-balance assay was performed with a combination of serum (D1-14: **A**–**D**, D2-44: **E**–**H**, D2-57: **I**–**L**, D3-46: **M**–**P**, D4-12: **Q**–**T** and D4-49: **U**–**X**) and four prototype viruses (DENV-1: Mochizuki strain; DENV-2: NGC strain; DENV-3: H87 strain; and DENV-4: H241 strain). The *blue*, *green*, *yellow* and *red* triangles, corresponding to Fig. 1A, indicate the hours after fever onset. The abscissa indicates the serum dilutions and the ordinate shows the numbers of infected cells (both expressed as log10). Each data point represents the average of two separate assays; error bars indicate the SDs. Dotted lines indicate the mean numbers of infected cells plus or minus three times the SD (mean ± 3SD), to define neutralizing or enhancing activity, respectively.

Dose-dependent neutralizing/enhancing activity curves were obtained against four prototype laboratory strains (DENV-1: Mochizuki; DENV-2: NGC; DENV-3: H87; and DENV-4: H241). Enhancing activity against at least one prototype strain was observe in all patients, of whom four patients (D2-44, D2-57, D4-12 and D4-49) showed remarkable shifts in the antibody levels against all four prototype DENVs (Fig. 2). Although patients D4-12 and D4-49 were currently infected with DENV-4, they displayed higher levels of cross-neutralization against heterologous DENV-2 and/or DENV-1 (Fig. 2Q,R,V). Similarly, patient D2-57, who was currently infected with DENV-2, showed cross-neutralization against DENV-4 (Fig. 2L). These results suggest that our assay system may be able to reveal the previous infection history in dengue patients by comparing their cross-neutralizing activities. On the other hand, shift of antibody level was limited in patients D1-14 and D3-46 (Fig. 2A–D,M–P). Patient D3-46 displayed strong neutralization against all serotypes (Fig. 2M–P), while patient D1-14 showed cross-reactive enhancing activities against prototypes DENV-1 and DENV-3 at 1:10 serum dilution (Fig. 2A,C). Based on these findings, it seems likely that a history of previous exposure to and infection by DENV affected the antibody response patterns in our patients.

### Evaluation of progeny virus titers by NAb/EAb balance assay

**Figure 3:**
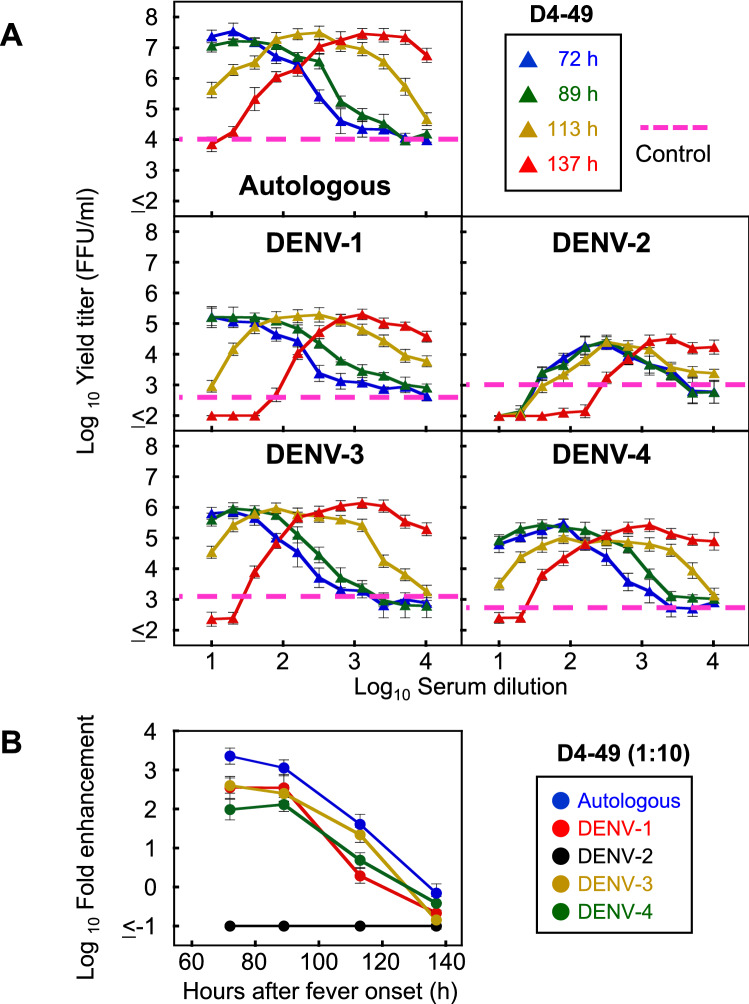
Progeny virus infectivity in the NAb/EAb balance assay. (**A**) Progeny virus titers obtained from the NAb/EAb-balance assay. The NAb/EAb-balance assay was conducted with combinations of patient D4-49 serum (twofold serial serum dilutions, starting from 1:10) and five DENV strains (the autologous, DENV-1: Mochizuki, DENV-2: NGC, DENV-3: H87 and DENV-4: H241 strain). Culture supernatants were harvested at 24 h after the mixture of serum, virus and K562 cells. The infective titers (FFU/ml) were determined on Vero cells. The *blue*, *green*, *yellow* and *red* triangles, corresponding to Fig. 1A, indicate the hours after fever onset. The abscissa indicates the serum dilutions and the ordinate shows the progeny virus titers (both expressed as log10). *Dotted lines* indicate the mean progeny virus titers calculated from four negative (no serum) controls. (**B**) Decreasing trend of ADE at low serum dilution with time progression. Fold enhancement was calculated from the infective progeny virus titers obtained in (**A**) (specifying data at 1:10 serum dilution), and was expressed in log10 as the increase in the progeny virus titer relative to the negative control. The fold enhancement was plotted as the ordinate against time (h) as the abscissa.

To evaluate the levels of progeny virus secreted from the infected cells using the NAb/EAb balance assay system, patient D4-49 was selected as a representative patient with a range of neutralizing and enhancing activities (Figs. 1 and 2). Culture supernatants were harvested from the infected cells 24 h after the set-up of the NAb/EAb-balance assay, and were titrated on Vero cells. The dose-dependent patterns of progeny virus titers (Fig. 3A) were roughly similar to those from the infected-cell counts (Figs. 1B and 2U–X), with minor differences at lower serum dilutions. Specifically, the defervescent sample (137 h) displayed lower yield titer than the control in all five strains at 1:10 serum dilution (Fig. 3A). Furthermore, fold enhancements were calculated from progeny virus titers at 1:10 serum dilutions (closer to the situation in vivo) (Fig. 3A), and were plotted as the ordinate against time (h) as the abscissae (Fig. 3B). The time-course patterns tended to decline in parallel, except for DENV-2. The autologous virus showed the higher fold enhancement (> 1000 fold on 72 h) than the other four prototype assay antigens. In contrast, patient D4-49 was currently infected with DENV-4, but this patient’s antibody reaction against DENV-2 was much higher than those against other serotypes, showing strong neutralizing activity against prototype DENV-2 at 137 h after fever onset (Fig. 2V). This finding suggests that the patient had been previously infected with DENV-2. Similar declining trends were observed in graphic data based on infected-cell counts as the ordinate (Supplementary Fig. S1).

### Relationship between the in vitro infective progeny virus and in vivo viral load

**Figure 4:**
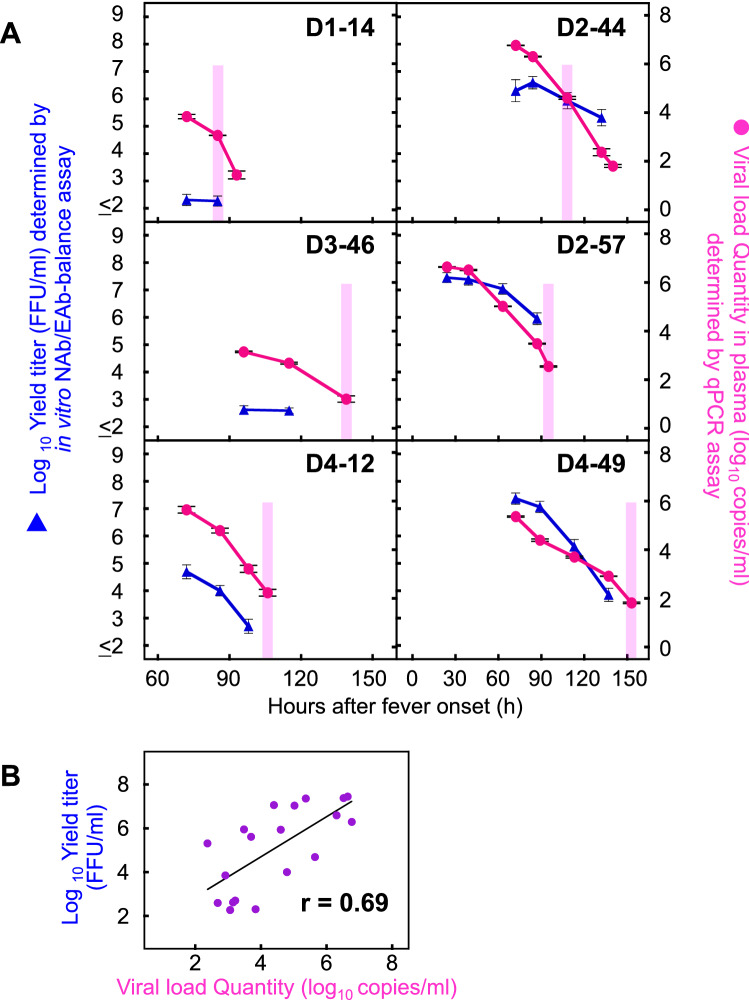
Relationship between in vitro ADE activity and in vivo viral load quantity. (**A**) The NAb/EAb-balance assay was conducted with a combination of serum diluted at 1:10 and the corresponding autologous virus. Culture supernatants were harvested at 24 h after the mixture of serum, virus and K562 cells. The infective titers were determined on Vero cells and plotted as the left ordinates (*blue triangles*: expressed as log10 FFU/ml). The number of viral RNA copies in plasma samples collected at same time points as well as one extra time point (D1-14: 93 h, D2-44: 140 h, D3-46: 139 h, D2-57: 95 h, D4-12: 106 h and D4-49: 153 h) were determined by real time RT-PCR, and plotted as the right ordinates (*red circles*: expressed as log10 copies/ml). The abscissae indicate time (h) after fever onset. *Pink shading* indicates the beginning of defervescence on the clinical observation. (**B**) Correlation between the ADE progeny virus titers and the viral RNA copy numbers. The correlation coefficient (r) was estimated for the individual progeny virus titers and qPCR values obtained in Fig. 4A (however, following samples were excluded; D1-14: 93 h, D2-44: 140 h, D3-46: 139 h, D2-57: 95 h, D4-12: 106 h and D4-49: 153 h). The abscissa and ordinate indicate the qPCR values and the progeny virus titers, respectively. Linear regression lines and r values are presented in the panel.

Using the same method described above, the infection-enhanced progeny virus titers were determined with the autologous combination (using 1:10 serum dilution) in the other five patients (D1-14, D2-44, D2-57, D3-46 and D4-12) (blue triangles in Fig. 4A). In addition, the quantity of viral RNA was directly determined from the corresponding clinical plasma samples (red circles in Fig. 4A). Both the ADE progeny virus titer (blue triangle) and viral RNA copy number (red circle) basically declined in parallel over time. Significant correlation was observed between the ADE progeny virus titers and the viral RNA copy numbers (r = 0.69; *P* < 0.05) (Fig. 4B). These results indicate that there might be positive correlations between (i) the in vitro progeny virus titers obtained from the NAb/EAb-balance assay and (ii) the in vivo viral quantity in the plasma. In contrast, no correlations were observed between the laboratory results (the intensity of ADE activity) and the clinical diagnosis (DF or DHF). Patients D2-57, D3-46, D4-12 and D4-49 deteriorated around the defervescent phase (*pink shading* in Fig. 4A), but the levels of progeny virus titers and viral load in D3-46 (DHF patient) were not as high as those in other DHF patients. Likewise, D2-44 showed DF manifestations, but the levels of progeny virus titers and viral load were as high as those of DHF patients.

## Discussion

In the present study, we analyzed the balance between neutralizing and enhancing activities in serum samples which were collected from dengue patients at multiple time points between the acute and defervescent phases (Figs. 1 and 2). Patient D3-46 showed DHF manifestations in spite of the strong neutralization against both autologous and prototype viruses, and the dose-dependent curve was not meaningfully shifted over 96–115 h in this patient (Figs. 1B and 2M–P, and Table 1). These findings suggest that the strong neutralization revealed by the in vitro antibody assays did not completely neutralize the autologous virus in vivo (Fig. 4A), which led to the development of severe disease. In contrast, DF patient D2-44 did not show severe manifestations, even though the ADE activity patterns of this patient were as high as those of the DHF patients (D2-57 and D4-49). Therefore, when samples from post-hospitalized patients were used, it was still difficult to predict disease outcomes by measuring the balance between neutralizing and enhancing activities. Although other factors might be involved in determining the immune correlation (such as the treatment conditions in the hospital, current infecting serotype and previous infection history, etc.), the most valuable factor (and also the most difficult) is the collection of samples in the pre-infection or pre-hospitalization period through prospective cohort studies^28–31^. In regard to the relationship between pre-infection sera and disease severity, Kliks et al. have reported that the level of virus yield induced by ADE was significantly higher in a severely symptomatic group than an asymptomatic one^22^.

The ADE activities shown by the low serum dilution (1:10) were gradually decreased over time (Fig. 3B and Supplementary Fig. S1). These results suggest that neutralizing antibodies were immediately produced after the infection (Table 1), and then the original enhancing activity might have been competitively suppressed by the appearance of neutralizing antibodies^25,27^. However, some cases did not show a decreasing trend in the heterologous combinations (Supplementary Fig. S1). For instance, patient D2-44 displayed the decreasing trend against DENV-2 (both autologous and NGC strains) and DENV-4, but exhibited an increasing or flat trend against DENV-1 and DENV-3 (Supplementary Fig. S1). This suggests that patient D2-44 may have had different antibody reactions to different serotypes. Therefore, monitoring the balance between NAb and EAb activities against all serotypes is important for the readiness upon secondary heterotypic infection in seropositive individuals. On the other hand, the opposite trend was observed in the high-serum dilution (1:2560)—that is, the ADE activities were basically increased over time (Supplementary Fig. S2). This suggests that the induced neutralizing antibodies still possess the potential ADE activity at sub-neutralizing doses.

The first approved dengue vaccine, Sanofi’s Dengvaxia, has been introduced into approximately 20 endemic countries since 2015^32^. However, WHO SAGE revised their recommendation to say that only seropositive individuals, who have previously been infected with DENV, can be inoculated with Dengvaxia^33^. Seronegative populations should not be vaccinated^34,35^, because a risk of vaccine-induced infection enhancement cannot be excluded^36–38^. Although there are several assay kits to detect dengue antibodies in a quantitative or qualitative manner (e.g., ELISA, immunochromatography assays, etc.), few rapid test kits are available to detect the functional antibody activity. Only an assay with the capacity to detect ADE activity would be able to provide a risk assessment for vaccine recipients. In the present study, five (D2-44, D3-46, D2-57, D4-12 and D4-49) of six patients were confirmed to be seropositive (classified as secondary infection). In contrast, patient D1-14 (classified as primary infection) showed no enhancing antibody activity against the autologous DENV-1, prototype DENV-2 and prototype DENV-4 strains (Figs. 1 and 2). This patient might show cross-reactive enhancing activities against the prototype DENV-1 and prototype DENV-3 strains (Fig. 2A,C). Thus, such individuals might consider to suspend the vaccination until their antibody levels are elevated sufficiently, if they have a plan to get Dengvaxia in the near future.

A previous study showed that ADE contributed to an increase in viremia in an animal model^39^. High viremia levels in dengue patients have also been reported to correlate with disease severity^6,9–13^, although the significance of the correlation is dependent on the disease day^14^. Since the viremia in humans is caused by secretion of the progeny viruses from the infected-host cells, the viral titers in the supernatant obtained from the in vitro NAb/EAb-balance assay might correlate to the clinical viral load. Interestingly, in the present study, significant positive correlations were observed between progeny virus titers (focus forming units [FFU]/ml) in the NAb/EAb-balance assay and in vivo viremia levels (copies/ml) in plasma samples (Fig. 4B). This result suggests that measurement of the infective progeny virus titers (or the infected cell counts) in the NAb/EAb-balance assay may enable us to predict the viral load in the subsequent DENV infection. Since patient D3-46 showed neutralizing activity rather than enhancing activity against autologous virus in the lower serum dilutions (Fig. 1B), the viral load quantity in this patient also might have been lower than those in other patients (Fig. 4A).

In conclusion, we revealed that the in vitro progeny virus titers obtained from an NAb/EAb-balance assay were significantly correlated with the viral load in patient plasma samples determined by qPCR assay. This suggests that the present NAb/EAb-balance assay might be used to predict the viral load that would result from a subsequent infection with DENV. However, we could not find a relationship between the ADE activity (viremia level) and disease severity during the early and defervescent phases. The major limitation of this study was that only six patients were enrolled, since the sample was limited to patients in whom the virus isolation was successful. Nonetheless, we believe that the analysis of the combination of autologous virus and serum is meaningful to understand the relationship between neutralization/ADE and disease outcome. Another limitation was that the present ADE experiments were performed using only K562 cells, which express Fc gamma receptor IIa but not Fc gamma receptor I. A multidirectional analysis using another cell line expressing Fc gamma receptor I may need to be performed to measure antibody-dependent neutralization and enhancement.

## Materials and methods

### Blood samples

The present study was conducted using the serum and plasma collected from confirmed-dengue patients at the Hospital for Tropical Diseases, Bangkok, Thailand, in a previous study^13^. The patients were diagnosed based on the World Health Organization (WHO) criteria of 1997^40^. The infection history (primary or secondary infection) of patients was determined based on the results of IgG and IgM antibody presence in acute sera using Panbio Dengue Duo Cassette (Abbott Inc., Chicago, IL) in accordance with the manufacturer’s protocol. Heat inactivation of sera was performed at 56 °C for 30 min. Six dengue patients were enrolled from whom clinical virus isolates were obtained. All subjects gave their informed consent for inclusion into the study before they participated. This study was conducted in accordance with the Declaration of Helsinki, and the protocol was approved by the Ethics Committee of the Faulty of Tropical Medicine, Mahidol University, Thailand (FTM ECF-019-04).

### Cells

African green monkey kidney Vero cells (CCL-81; American Type Culture Collection, Manassas, VA) were cultivated in Eagle’s minimum essential medium supplemented with 10% fetal bovine serum (FBS) and 60 µg/mL kanamycin^41^. Human erythroleukemia K562 cells were cultivated in RPMI 1640 medium supplemented with 10% FBS, 100 units/mL penicillin, and 100 µg/mL streptomycin^42^. All cell lines were cultivated in a humidified atmosphere of 5% CO_2_: 95% air at 37 °C.

### Virus isolation and typing

Vero cells were inoculated with the diluted patient sera (1:10) collected in the acute phase, and then incubated at 37 °C for 7 days. Within five blind passages, viral RNA was extracted from the supernatant of the infected cells, and the serotype was determined by PCR using type-specific primers following a previous report^43^.

### Viruses

Six clinical isolates (1 strain of DENV-1, 2 of DENV-2, 1 of DENV-3, and 2 of DENV-4) and four prototype lab strains (DENV-1: Mochizuki strain; DENV-2: New Guinea C [NGC] strain; DENV-3: H87 strain; and DENV-4: H241 strain)^44^ were used in this study. The culture supernatants harvested from the infected Vero cells were used as live virus sources for the neutralization test and the antibody assay measuring the balance between neutralizing and enhancing activities, and as antigens for ELISA (see below).

### Neutralization test against autologous DENV isolate

The Vero cell focus reduction neutralization test of the serum samples was performed with autologous DENV isolates, essentially as described previously^45^. Briefly, mixtures of the virus and two-fold serial dilutions of serum samples (starting from 1:10) were incubated at 4 °C overnight. Vero cell monolayers were inoculated with the virus–antibody mixture and incubated at 37 °C for 3 days. After fixation and immunostaining using 4G2 antibody, the foci were counted. The neutralizing activities are expressed as percentages of focus reduction calculated relative to the results for virus controls without test samples.

### ELISA for measuring antibodies to autologous DENV isolate

Antibody levels in serum samples were measured by a conventional ELISA as described previously^42^. Briefly, 96-well microplates sensitized with each autologous DENV were incubated serially with the corresponding two-fold serial dilutions of serum samples (starting from 1:100), anti-human IgG (H + L) alkaline phosphatase conjugate (Promega, Madison, WI), and then p-nitrophenyl phosphate. The end-point titers were expressed as the maximum serum dilution that displayed an optic density (OD) value of ≥ 0.3.

### Antibody assay for the balance between neutralizing and enhancing activities (NAb/EAb-balance assay)

The NAb/EAb-balance assay was conducted using semi-adherent K562 cells as described previously^26^. Briefly, serial dilutions of sera (starting from 1:10 dilution) were mixed with each DENV strain in a poly-L lysine-coated 96-well microplate and incubated at 37 °C for 2 h. K562 cells (1 × 10^5^ cells per well) were then added to the mixtures and incubated at 37 °C for 2 days. After immunostaining (see below), the infected cells were counted. The cut-off values for neutralizing and enhancing activities were calculated from the means ± three standard deviations (SD) of infected cell counts obtained with eight negative controls adjusted for approximately 100 infected cells. When the number of infected cells was higher than the mean +  3SD, it was defined as enhancing activity. In contrast, when the infected cell number was lower than the mean − 3SD, it was defined as neutralizing activity.

In addition, enhancing activity was also evaluated by titrating the progeny virus infectivity in the culture supernatant of the infected cells. The supernatants were harvested at 24 h after the inoculation, and their titers were determined on Vero cells by counting infectious foci after immunostaining (see below) and expressed as FFU/ml.

### Immunostaining

Immunochemical staining was performed essentially as described previously^41^. Briefly, cells were fixed with acetone/methanol (1:1) and incubated serially with a mouse monoclonal antibody D1-4G2 (flavivirus group cross-reactive) purchased from American Type Culture Collection (Manassas, VA), biotinylated anti-mouse IgG, ABC (avidin-biotinylated peroxidase complex) reagent, and VIP substrate (Vector Laboratories, Burlingame, CA).

### Quantification of the viral RNA copy number in plasma samples

The viral RNA copy number in plasma samples was determined by following a previous study^13^. Briefly, viral RNA was extracted from 70 μl plasma using a QIAamp Viral RNA Mini Kit (Qiagen, Hilden, Germany) according to the manufacturer’s protocol, then subjected to quantitative real-time RT-PCR using a One-Step SYBR PrimeScript RT-PCR kit II (Takara Bio, Japan). The PCR mixture was mixed with 2 μl of extracted RNA and DENV-specific primer^46^ before running on a CFX96TM real-time PCR cycler (BioRad, Hercules, CA, USA) under cycle conditions of 42 °C for 5 min, 95 °C for 10 s followed by 45 cycles of 95 °C for 5 s, 55 °C for 30 s and 72 °C for 30 s. The viral load quantity was determined by linear regression of the cycle threshold value against the known viral titers quantified by focus forming unit assay.

### Statistical analysis

Correlation coefficients were estimated on the basis of the Pearson product-moment correlation coefficient (r). A probability (*p*) less than 0.05 was considered statistically significant.

## Supplementary Information

Supplementary Information.
