# Supplementary material for: Antibody-dependent enhancement representing in vitro infective progeny virus titer correlates with the viremia level in dengue patients
Source: Sci Rep. 2021 Jun 11;11:12354. doi: 10.1038/s41598-021-91793-0 (PMC8196181; doi:10.1038/s41598-021-91793-0)
Supplement: Supplementary file 1 — Supplementary Information. [file 41598_2021_91793_MOESM1_ESM.pdf]

Antibody-dependent enhancement representing *in vitro* infective progeny virus titer correlates with the viremia level in dengue patients

Atsushi Yamanaka,[1,2,*] Hisham Ahmed Imad,[3] Weerapong Phumratanaprapin,[3] Juthamas Phadungsombat,[1] Eiji Konishi,[4] and Tatsuo Shioda[1,2,5]

[1]*Mahidol-Osaka Center for Infectious Diseases, Faculty of Tropical Medicine, Mahidol University, 420/6 Ratchawithi Road, Ratchathewi, Bangkok 10400, Thailand.*

[2]*Mahidol-Osaka Center for Infectious Diseases, Research Institute for Microbial Diseases, Osaka University, 3-1 Yamada-oka, Suita, Osaka 565-0871, Japan.*

[3]*Department of Clinical Tropical Medicine, Faculty of Tropical Medicine, Mahidol University, 420/6 Ratchawithi Road, Ratchathewi, Bangkok 10400, Thailand.*

[4]*BIKEN Endowed Department of Dengue Vaccine Development, Faculty of Tropical Medicine, Mahidol University, 420/6 Ratchawithi Road, Ratchathewi, Bangkok 10400, Thailand.*

[5]*Department of Viral Infections, Research Institute for Microbial Diseases, Osaka University, 3-1 Yamada-oka, Suita, Osaka 565-0871, Japan.*

Supplementary Table S1. ELISA antibody titers against the autologous virus

		Patient ID					
	hours	D1-14	D2-44	D2-57	D3-46	D4-12	D4-49
Period after fever onset	24	-	-	1:100	-	-	-
	39	-	-	1:200	-	-	-
	63	-	-	1:200	-	-	-
	72	1:100[a]	1:200	-	-	1:400	1:40
	84	-	1:800	-	-	-	-
	85	1:100	-	-	-	-	-
	86	-	-	-	-	1:800	-
	87	-	-	1:400	-	-	-
	89	-	-	-	-	-	1:80
	96	-	-	-	1:12,800	-	-
	98	-	-	-	-	1:800	-
	108	-	1:1,600	-	-	-	-
	113	-	-	-	-	-	1:320
	115	-	-	-	1:25,600	-	-
	132	-	1:1,600	-	-	-	-
	137	-	-	-	-	-	1:2,560
Infection history		Primary	Secondary	Secondary	Secondary	Secondary	Secondary

[a] The end-point titer was expressed as the maximum serum dilution that displayed an optic density (OD) value of $\geq$0.3.

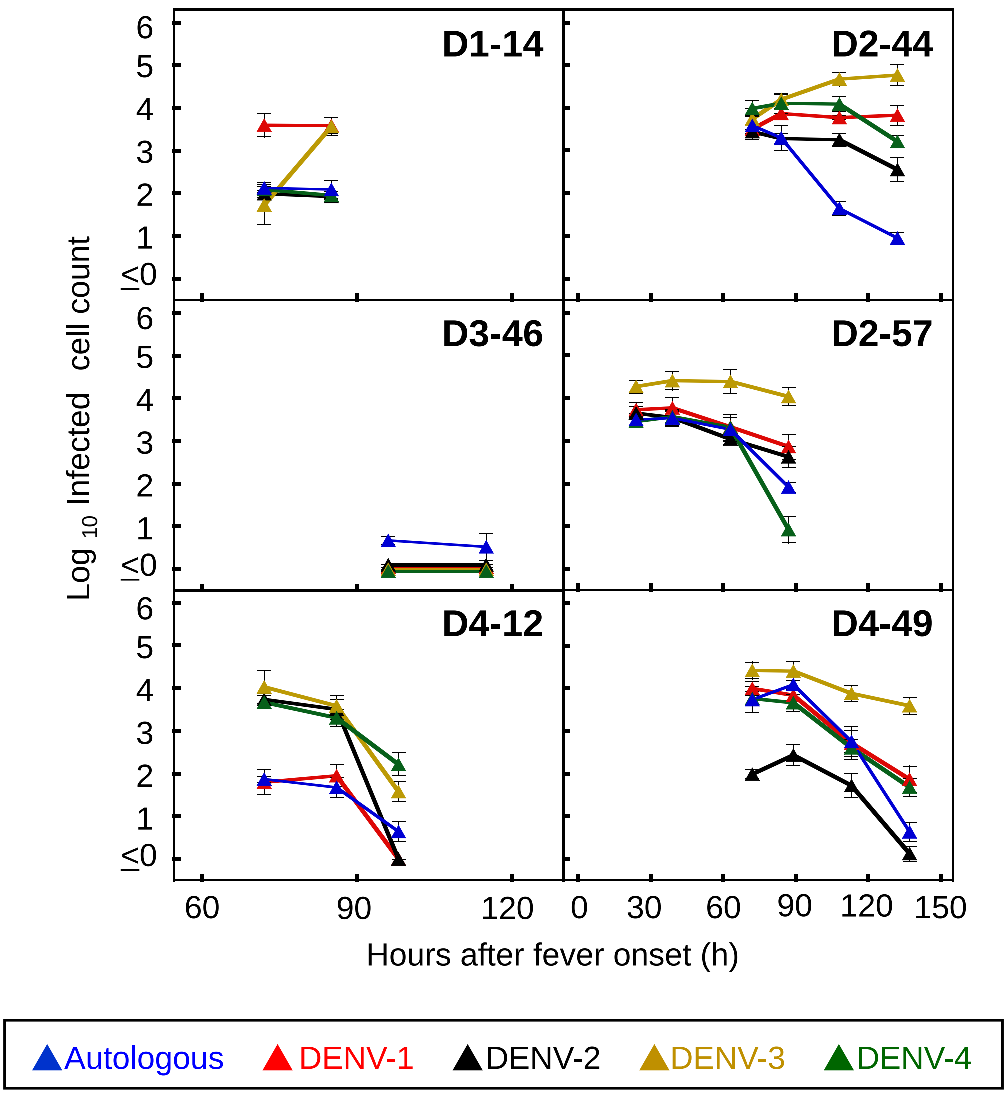

Supplementary Figure S1. Transition of the NAb/EAb balance activity in a low serum dilution (1:10) with time progression. The infected cell counts (specifying data at 1:10 serum dilution) obtained from Figs. 1B and 2 were plotted as the ordinate (expressed as log10) against time (h) as the abscissa.

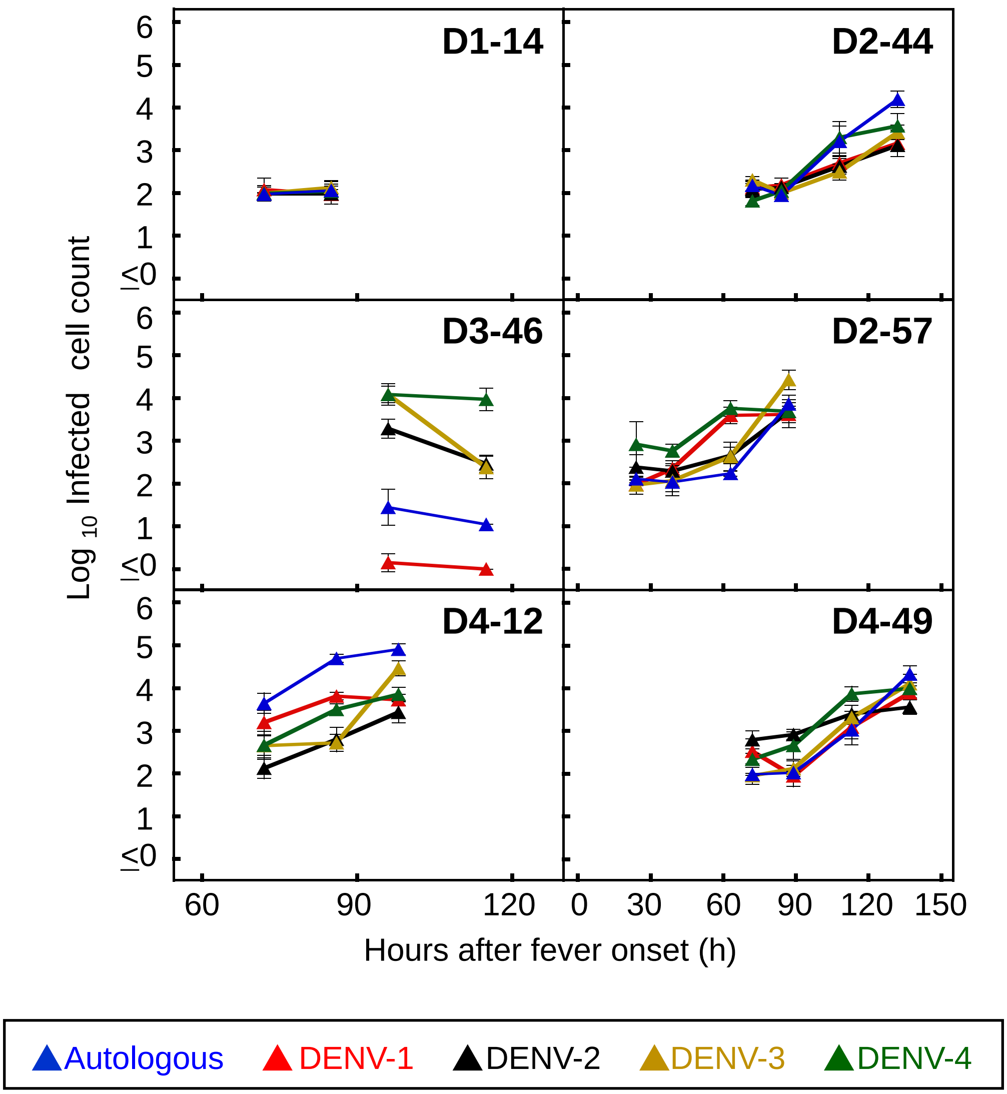

Supplementary Figure S2. Transition of the NAb/EAb balance activity in a high serum dilution (1:2560) with time progression. The infected cell counts (specifying data at 1:2560 serum dilution) obtained from Figs. 1B and 2 were plotted as the ordinate (expressed as log10), and plotted with time (h) as the abscissa.
